# SERPINB2 is a novel indicator of stem cell toxicity

**DOI:** 10.1038/s41419-018-0748-x

**Published:** 2018-06-20

**Authors:** Na-Hee Lee, Ara Cho, Se-Ra Park, Jin Woo Lee, Park Sung Taek, Chan Hum Park, Yoon-Hyeong Choi, Soyi Lim, Min-Kwan Baek, Dong Young Kim, Mirim Jin, Hwa-Yong Lee, In-Sun Hong

**Affiliations:** 1 Department of Health Sciences and Technology, GAIHST, Gachon University, Incheon, 21999 Republic of Korea; 2 Department of Molecular Medicine, School of Medicine, Gachon University, Incheon, 406-840 Republic of Korea; 3 Department of Health Sciences and Technology, SAIHST, Sungkyunkwan University, Seoul, Republic of Korea; 4 Department of Obstetrics and Gynecology, Hallym University Kangnam Sacred Heart Hospital, Seoul, Republic of Korea; 5 Department of Otolaryngology-Head and Neck Surgery, Chuncheon Sacred Heart Hospital, Hallym University College of Medicine, Chuncheon, Republic of Korea; 6 Department of Preventive Medicine, Gachon University Graduate School of Medicine, Incheon, Republic of Korea; 7 Department of Obstetrics and Gynecology, Gachon University Gil Medical Center, Incheon, Republic of Korea; 8 Department of Otolaryngology-Head and Neck Surgery, Gil Medical Center, Gachon University School of Medicine, Incheon, Republic of Korea; 9 College of Medicine, Gachon University, Incheon, Republic of Korea; 10 Department of Biomedical Science, Jungwon University, 85 Goesan-eup,Munmu-ro, Goesan-gun, Chungcheongbuk-do 367-700 Republic of Korea

## Abstract

The toxicological evaluation of potential drug candidates is very important in the preclinical phase of drug development. Toxic materials may cause serious decline in stem cell function and loss of stemness. Indeed, we found that toxic exposure more profoundly suppressed the growth of stem cells than terminally differentiated fibroblasts. Importantly, toxic exposure suppressed stem cell migration and multi-lineage differentiation potential in vitro and in vivo. Moreover, early-response genes involved in stem cell properties such as self-renewal and differentiation capabilities can be used as specific markers to predict toxicity. In the present study, we also identified a labile toxic response gene, SERPINB2, which is significantly increased in response to various toxic agents in human stem cells in vitro and in vivo. Consistently, self-renewal, migration, and multi-lineage differentiation potential were markedly decreased following SERPINB2 overexpression. To the best of our knowledge, this is the first study to focus on the functions of SERPINB2 on the regenerative potential of stem cells in response to various existing chemicals, and the findings will facilitate the development of promising toxicity test platforms for newly developed chemicals.

## Introduction

The current evaluation methods for a drug’s safety largely rely on non-human animal-based platforms. However, even advanced animal-based platforms do not appropriately mimic extremely complex human physiology^1^. The most famous example of a drug that was considered safe after animal tests but later proved to have devastating effects in human trials is thalidomide, which had no effect on fetal development in experimental animal but which induced severe developmental defects in humans^2^. While human tumor-derived or engineered cell-based systems have some advantages for evaluation, they also have genomic abnormalities and do not reflect the complex physiology of real tissues^3^. Stem cells are capable of differentiating into multiple cell types and are involved in the long-term maintenance of tissue homeostasis^4^. Interestingly, due to their varying states of differentiation, stem cells can respond differently to the same chemical exposure, and thus differential toxic effects might be expected^5^. In this context, stem cell-based screening platforms can provide valuable information on newly developed chemicals that are not normally detected by other somatic cell-based screening system.

Importantly, early changes in the gene-expression profile mediated by exposure to toxic materials are more likely to indicate the initiation of toxic processes than are late-stage events, thus providing more sensitive and accurate markers of early toxic events^6^. Toxic materials may cause serious decline in stem cell function and loss of stemness^7^. Therefore, early-response genes involved in stem cell properties, such as self-renewal and differentiation capabilities, can be used as specific markers to predict toxicity. Our current understanding of gene expression profiles for predicting toxic responses is very limited. Therefore, to identify the early-response genes associated with possible toxic effects, we compared the high-throughput DNA microarray and RNA sequencing gene expression profiles of human stem cells treated with well-known standard toxic compound (dioxin) to those of non-treated cells. Several previous studies have investigated the effects of dioxin on various types of animal stem cells, including mouse embryonic^8, 9^, mouse hematopoietic^10^, and rodent bone marrow^11^ stem cells, suggesting the reliability of dioxin as a standard toxic compound for stem cell toxicity.

Among the genes that were analyzed, we observed significant positive correlation between toxic exposure and enhanced SERPINB2 expression. SERPINB2, also known as plasminogen activator inhibitor type 2 (PAI-2), is highly increased in response to the classic terminal cellular differentiation agent retinoic acid in multiple cell types, such as epidermal keratinocytes^12^, peripheral blood mononuclear cells^13^, and promyelocytic leukemia cells^14, 15^, indicating that SERPINB2 is involved in the process of cell differentiation. Indeed, other studies demonstrated that enhanced SERPINB2 levels reduce cell proliferation and are associated with the increased expression of differentiation-specific markers^16–18^. Furthermore, SERPINB2 has been identified as one of the synergistically dysregulated genes that stimulate leukemia stem cell proliferation and survival^19^. These results suggested that SERPINB2 could serve as a sensitive marker for predicting toxic responses such as defective cell proliferation or differentiation to various chemicals.

In conclusion, we demonstrate here for the first time that SERPINB2 expression is significantly increased in response to various toxic agents in stem cells in vitro and in vivo. More strikingly, we also reveal that SERPINB2 has the capacity to regulate the proliferation and differentiation potential of human stem cells, suggesting that SERPINB2 can be used as one of the possible markers for predicting toxicity. Currently, the number of hazardous materials is growing as a result of rapid industrialization, warranting a better screening platform for predicting toxicity. Our stem cell-based screening platforms can provide valuable information on newly developed chemicals that are not normally detected by other somatic cell-based systems by combining the toxic response gene SERPINB2.

## Results

### Toxic exposure suppresses the self-renewal and differentiation potential of stem cells in vitro and in vivo

**Fig. 1:**
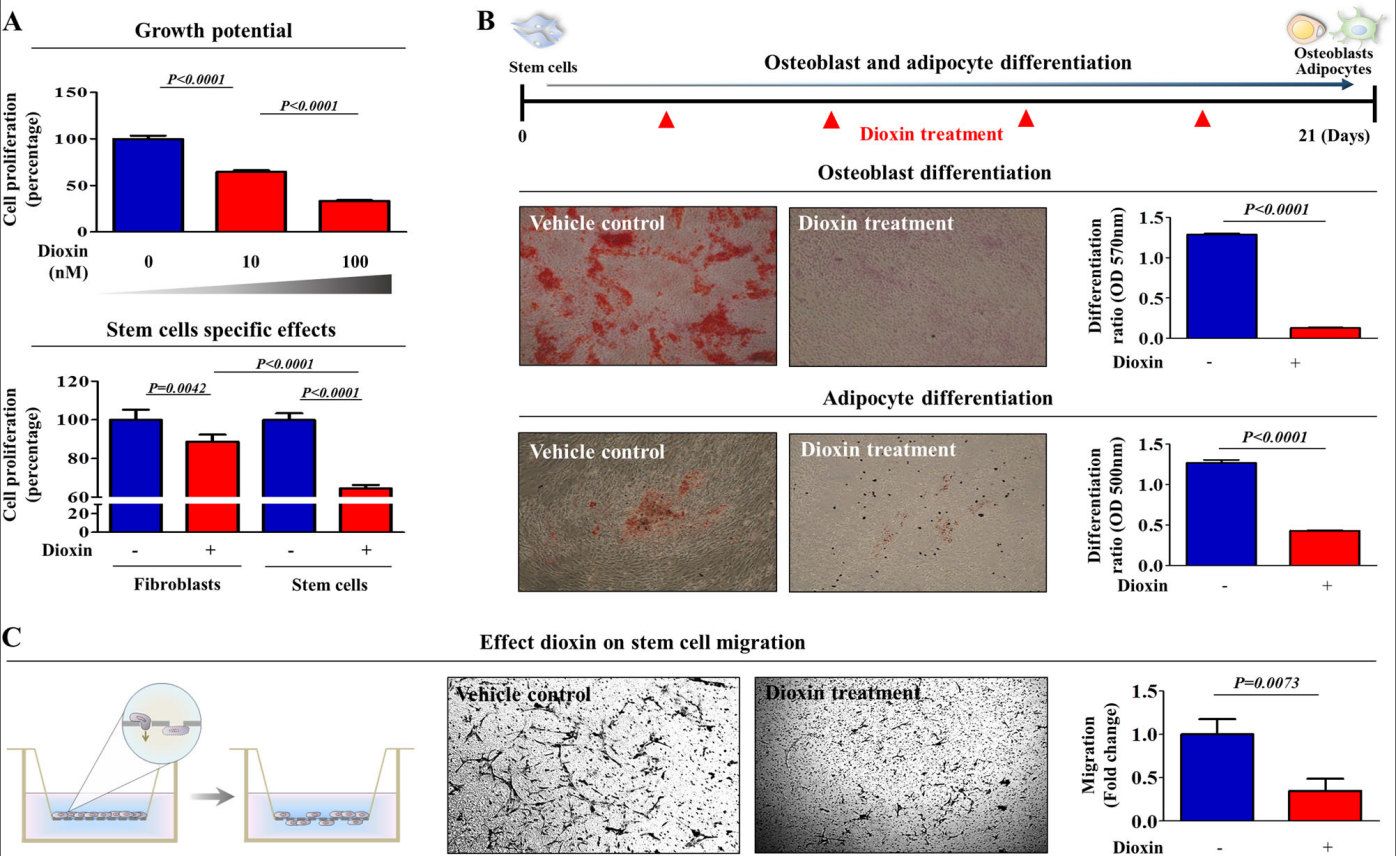
The effects of dioxin on the various functions of stem cells in vitro. The inhibition of cell viability by dioxin treatment for 72 h was determined via an MTT assay in both stem cells and fibroblasts. The cell viability (%) was calculated as the percent of the vehicle control (**a**). Schematic representation described the experimental protocol for differentiation and treatment. Confluent stem cells were cultured for 21 days in osteogenic or adipogenic medium with or without dioxin (10 nM). The effect of dioxin on osteoblast or adipocyte differentiation was determined by alizarin red or oil red O staining, respectively. Relative quantification of calcium mineral content or lipid droplet formation was determined by absorbance measurements at 570 nm or 500 nm, respectively (**b**). The effect of dioxin on the MSC migration ability was evaluated using a transwell migration assay. Dioxin treatment significantly decreased MSC migration across the membrane compared with the negative controls (**c**). The results are presented as the mean ± SD from at least three independent experiments

**Fig. 2:**
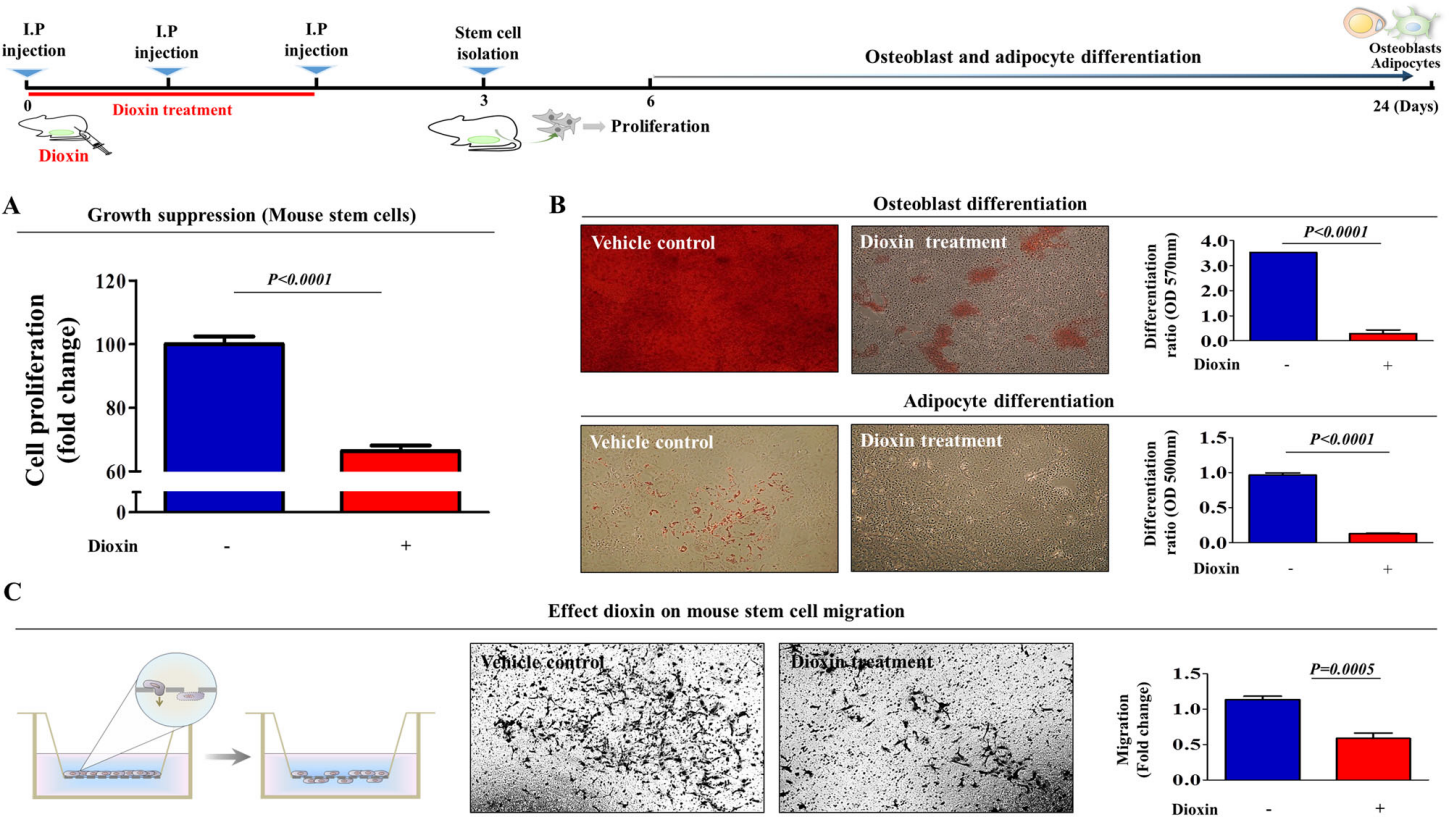
Toxic exposure affects the in vivo growth and differentiation potential of stem cells. Schematic representation described the experimental protocol for dioxin treatment in vivo. Mice were treated with dioxin (15 µg/kg, intraperitoneally, three times daily, for 3 days) or vehicle (DMSO), and then after 3 days stem cells from mouse adipose tissue were isolated. The effect of dioxin on cell viability was determined via an MTT assay (**a**). Stem cells derived from mouse adipose tissues were cultured in osteogenic or adipogenic differentiation medium for 21 days without additional dioxin treatment. The effect of dioxin on osteoblast or adipocyte differentiation in vivo was determined by alizarin red or oil red O staining, respectively (**b**). The effect of dioxin on the migration ability of mouse adipose tissue-derived MSCs in vivo was evaluated using a transwell migration assay (**c**). The results are presented as the mean ± SD from three independent experiments

**Fig. 3:**
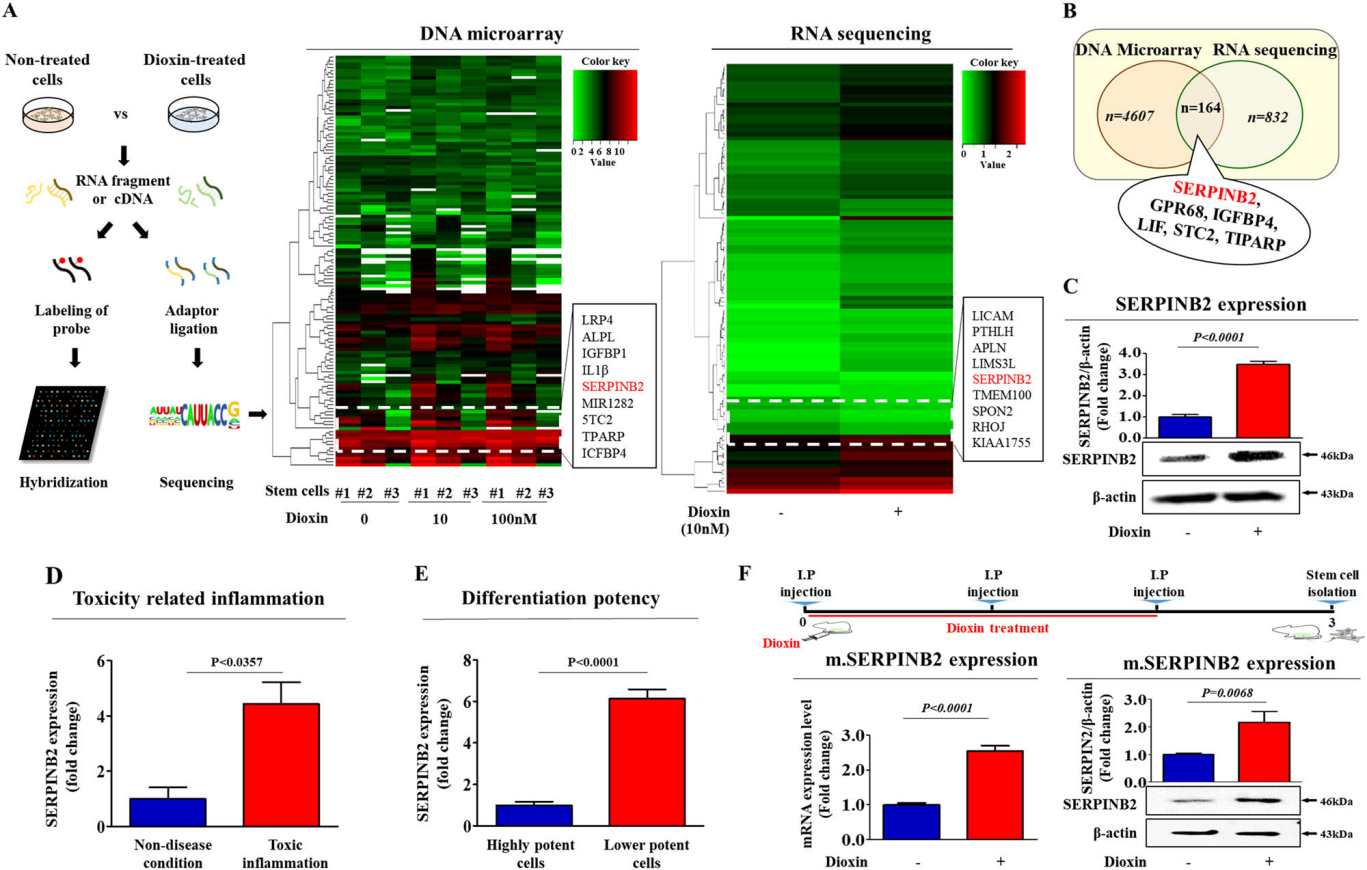
Dioxin stimulates SERPINB2 expression in stem cells in vitro and in vivo. The data from both large-scale DNA microarray and RNA sequencing are presented as a heatmap of differentially expressed genes in non-treated stem cells compared to those treated with dioxin; decreased (green) or increased (red) expression compared to the mean mRNA expression are indicated (**a**). Among the genes that were analyzed, we observed a positive correlation between dioxin exposure and enhanced SERPINB2 expression in stem cells (**b**). Western blotting was used to verify enhanced SERPINB2 expression in DNA microarray and RNA sequencing (**c**). Clinical big data were analyzed using the Seiber dataset (GSE43996 and GSE9452) from ‘R2: Genomics Analysis and Visualization Platform (http://r2.amc.ml)’. The gene datasets were filtered by SERPINB2 expression profiles and the differentiation potency of stem cells (**d**) or toxicity-related diseases (**e**). Mice were treated with dioxin (15 µg/kg, intraperitoneally) or vehicle (DMSO), and then stem cells from the adipose tissue of the mice were isolated and expanded as described in the materials and methods section. Real-time PCR and western blotting were performed to confirm the increased SERPINB2 expression in vivo by dioxin exposure (**f**). β-actin was used as an internal control. The results represent the means ± SD from three independent experiments

**Fig. 4:**
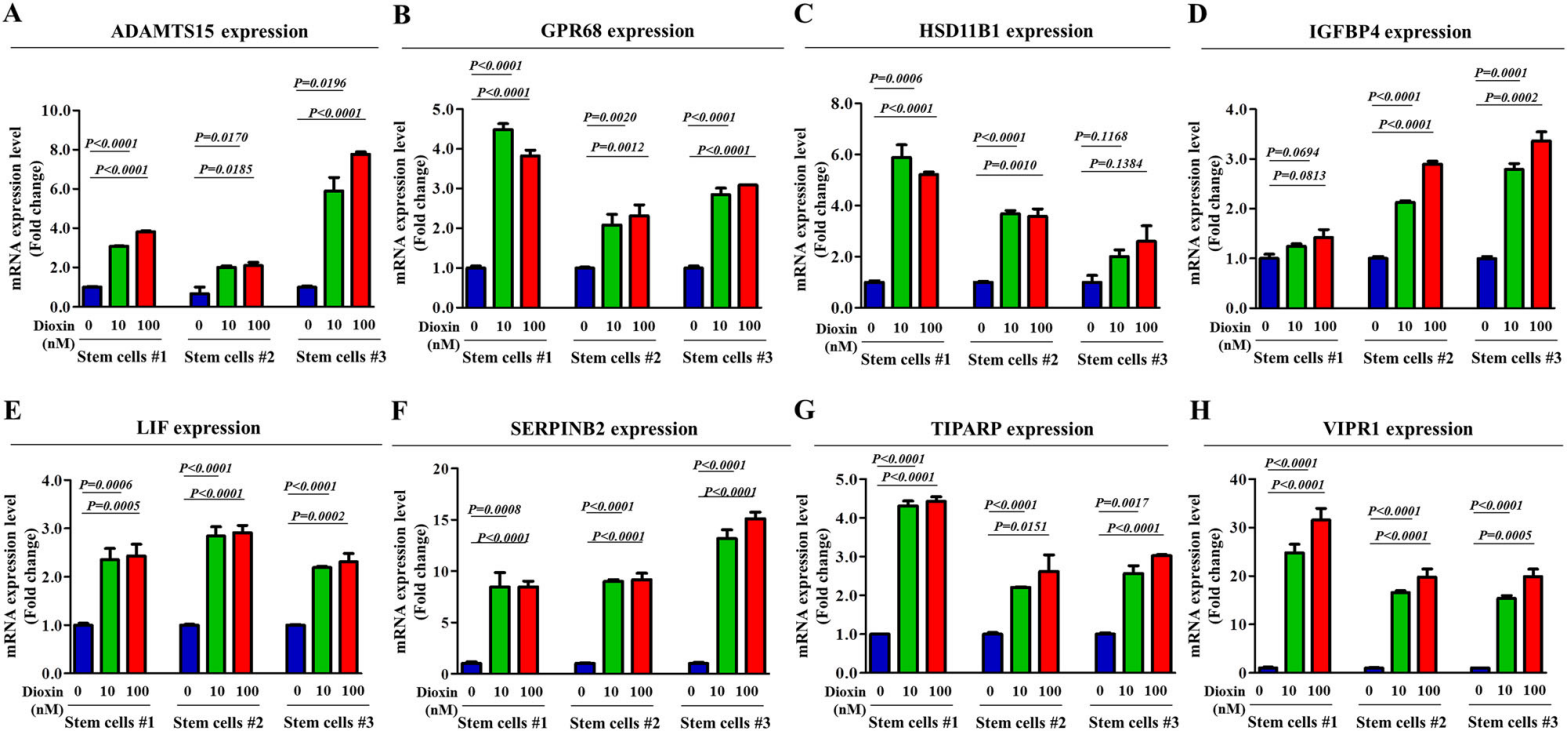
Effects of dioxin on the expression of various potential response genes. The stimulatory effect of dioxin on the expression levels of potential response genes, such as ADAMTS15, GPR68M, HSD11B1, IGFBP4, LIF, SERPINB2, TIPARP, and VIPR1, were assessed in stem cells isolated from three different individuals using real-time PCR (**a**–**h**). The results represent the means ± SD from three independent experiments

To generate mesenchymal stem cell (MSC) lines for high-throughput screening, we isolated MSCs from human umbilical cord blood (UCB) using our standard method (Supple. Figure 1A) and then characterized the biological properties of UCB-derived MSCs by combining various positive and negative MSC surface markers (Supple. Figure 1B). Their ability to differentiate into various tissue lineages was confirmed by inducing osteoblast and adipocyte differentiation in vitro (Supple. Figure 1C). Standard toxic compound (dioxin) was chosen from the list of top-ranked compounds according to common hazardous material classification of five authorities, including the International Agency for Research on Cancer (IARC), Association Advancing Occupational and Environmental Health (ACGIH), National Toxicology Program (NTP), US Environmental Protection Agency (US EPA), and European Chemicals Agency (ECHA). A schematic summary of the test compound selection is described in Supple. Figure 2A. We found that dioxin (Supple. Figure 2B) exposure more profoundly affected the growth of stem cells than terminally differentiated dermal fibroblasts (Fig. 1a) without affecting the levels of stem cell-specific markers such as CD44, CD73, and CD105 (Supple. Figure 3), suggesting that stem cells are likely to be more sensitive to toxic exposure than terminally differentiated somatic cells. Toxic exposure also significantly suppressed multi-lineage differentiation potential toward osteoblasts and adipocytes in vitro (Fig. 1b). A transwell migration assay demonstrated the inhibitory effect of toxic materials on the migration ability of stem cells in vitro (Fig. 1c). To further confirm whether toxic exposure affects the in vivo growth and differentiation potential of stem cells, we treated mice with dioxin and isolated stem cells from adipose tissue. Then, adipose tissue-derived mouse stem cells were differentiated to osteoblasts and adipocytes in inductive medium. Toxic exposure consistently suppressed stem cell growth (Fig. 2a) and differentiation into osteoblasts and adipocytes (Fig. 2b) in vivo. A transwell migration assay demonstrated the inhibitory effect of toxic exposure on the migration ability of stem cells in vivo (Fig. 2c). However, these inhibitory effects did not bear any clear relation to the apoptotic processes and phenotypes, including the Annexin V-positive population (Supple. Figure 4A), caspase-3 activities (Supple. Figure 4B), PAPR expression (Supple. Figure 4C), and DNA fragmentation (Supple. Figure 4D).

### Aberrant activation of SERPINB2 in response to toxic exposure in stem cells

**Fig. 5:**
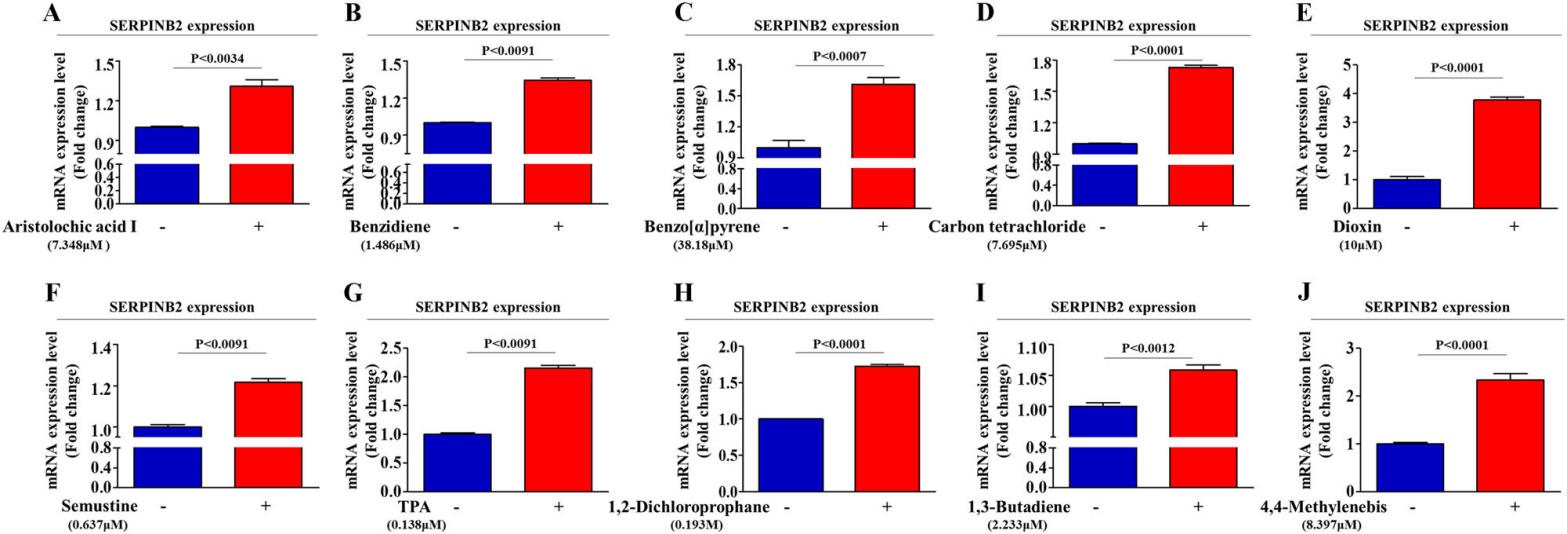
Effects of multiple test substances on SERPINB2 expression levels in stem cells. The stimulatory effect of multiple test substances on SERPINB2 expression levels in stem cells was assessed by real-time PCR (**a**–**j**). The results represent the means ± SD from three independent experiments

We compared differential gene expression in toxicant-treated stem cells and non-treated stem cells to identify potential target genes responsive to toxic materials using two large-scale gene expression analysis methods, RNA sequencing and DNA microarray. We identified a group of genes with levels affected by toxic exposure (Fig. 3a) and then verified their expression using real-time PCR (Fig. 4a–h). Among the genes that were analyzed, we observed a positive correlation between toxic exposure and enhanced SERPINB2 expression (Fig. 3b and Fig. 4f). Western blotting was used to further confirm altered SERPINB2 expression in the toxicant-exposed stem cells (Fig. 3c). To further confirm whether SERPINB2 expression was enhanced by loss of the differentiation potential or toxicity-related diseases, we additionally analyzed clinical big data using the Seiber dataset (GSE43996 and GSE9452) from ‘R2: Genomics Analysis and Visualization Platform (http://r2.amc.ml)’. The gene datasets were filtered by SERPINB2 expression profiles and the differentiation potency of stem cells or toxicity-related diseases. The results revealed a strong relationship between significantly increased SERPINB2 expression and toxicity-related inflammatory disease (Fig. 3d) or deceased differentiation potency (Fig. 3e). Additionally, to confirm the effects of toxic exposure on SERPINB2 expression in vivo, we treated mice with dioxin and isolated stem cells from the mice adipose tissue. Then, the SERPINB2 expression levels were analyzed by real-time PCR and western blotting. Consistently, dioxin exposure stimulated SERPINB2 expression in adipose-derived mouse stem cells (Fig. 3f). To further determine whether SERPINB2 can serve as a “universal” marker rather than a specific response gene to a certain chemical (dioxin) for predicting toxic responses to any types of toxic material, SERPINB2 expression profiles were investigated with various types of known toxic substances. The approximate IC_50_ values of multiple test substances were determined using a dose-response curve (Supple. Figure 5). Significantly enhanced SERPINB2 expression levels were detected with three of the tested toxic substances, and moderately increased levels were also detected with three other substances. However, four test substances rarely increased SERPINB2 expression in human stem cells (Fig. 5). These resultswarrantfurther prospective studies to verify the reliability of SERPINB2 as a universal marker for predicting toxicity.

### SERPINB2 overexpression reduces the self-renewal capacity and differentiation potential of stem cells

**Fig. 6:**
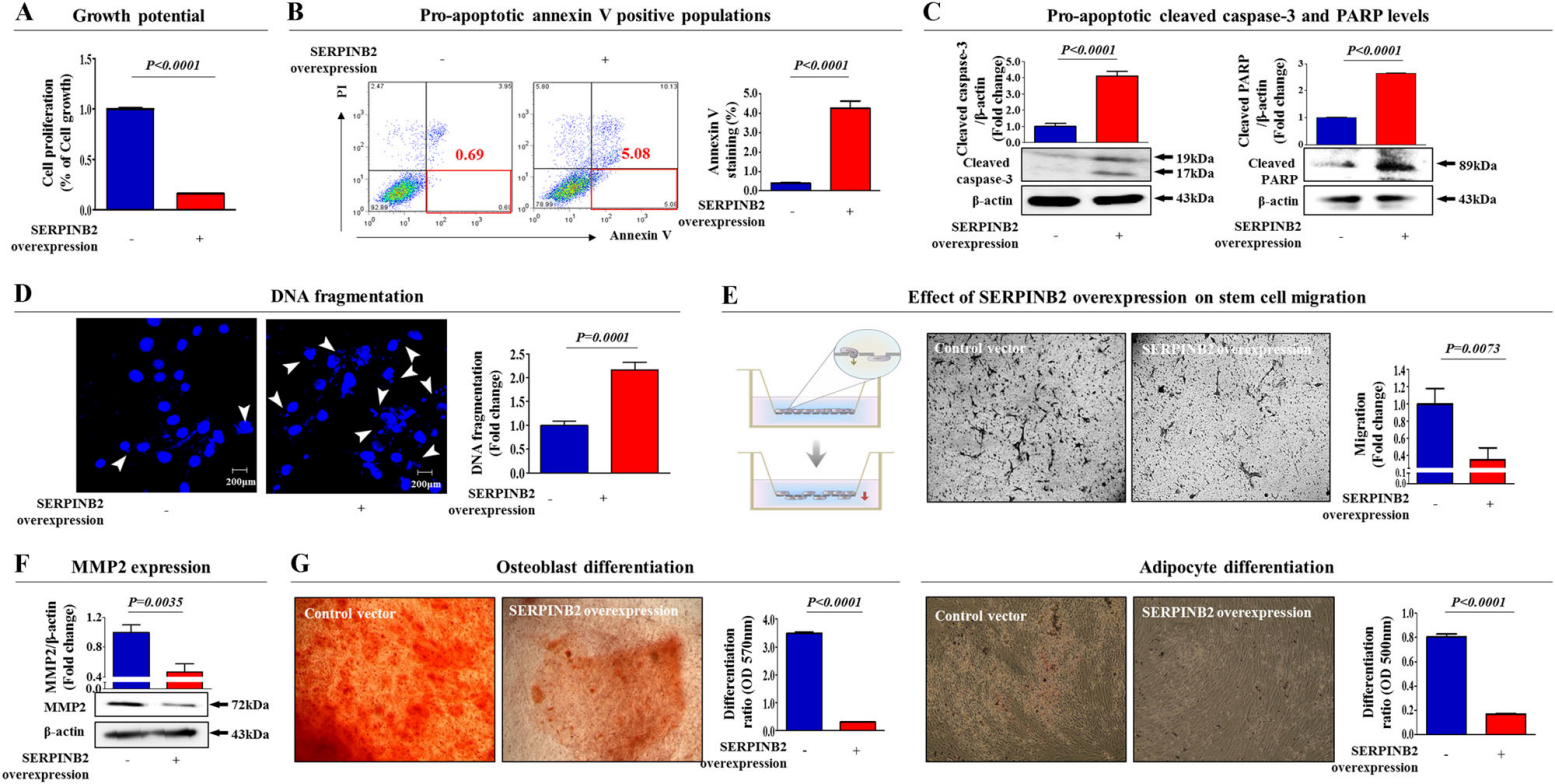
The effects of overexpression of SERPINB2 on the various functions of stem cells. Transfection of MSCs with a SERPINB2 vector led to a significant decrease in the number of cells compared with transfection using a control vector (**a**). SERPINB2 overexpression-mediated cytotoxicity was evaluated by flow cytometry using PE-labeled Annexin-V (**b**). Elevated levels of caspase-3 fragment following SERPINB2 overexpression were assessed by western blotting (**c**). SERPINB2 overexpression-mediated apoptotic DNA fragmentation and condensation were visualized using DAPI staining (**d**). The effects of SERPINB2 overexpression on the cell migration ability were evaluated using a transwell migration assay (**e**) and by western blotting using an MMP2 antibody (**f**). The effect of SERPINB2 overexpression on osteoblast or adipocyte differentiation was determined by alizarin red or oil red O staining, respectively. Relative quantification of the calcium mineral content or lipid droplet formation was determined by absorbance measurements at 570 nm or 500 nm, respectively (**g**). β-actin was used as an internal control. The results represent the means ± SD from three independent experiments

**Fig. 7:**
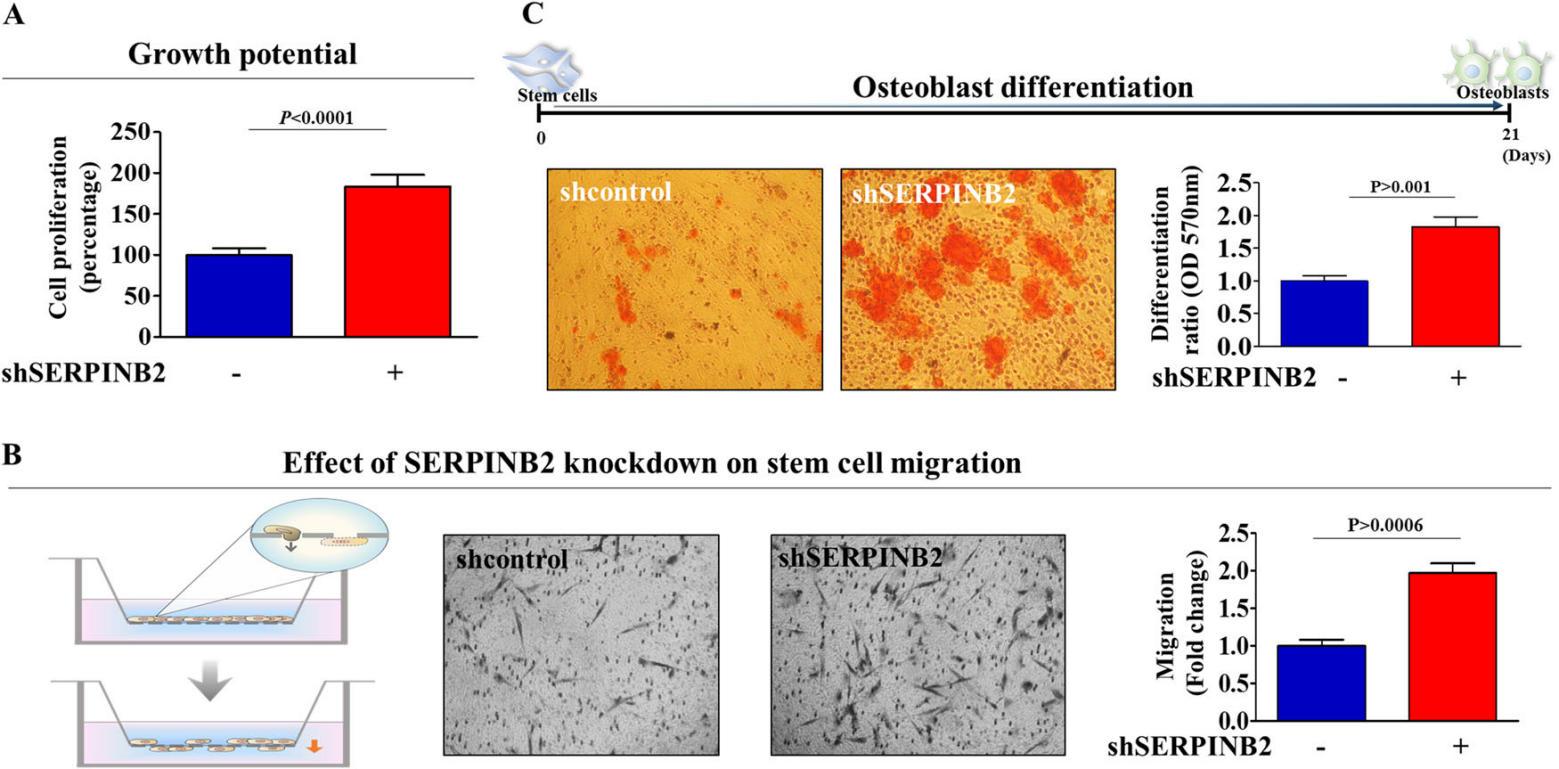
The effects of knockdown of SERPINB2 on the various functions of stem cells. Transfection of MSCs with a shSERPINB2 led to an increase in the number of cells compared with transfection using a control shRNA (**a**). The effects of SERPINB2 knockdown on the cell migration ability were evaluated using a transwell migration assay (**b**). The effect of SERPINB2 knockdown on osteoblast differentiation was determined by alizarin red staining. Relative quantification of the calcium mineral content or lipid droplet formation was determined by absorbance measurements at 570 nm (**c**). The results represent the means ± SD from three independent experiments

To confirm whether SERPINB2 can regulate the self-renewal capacity and differentiation potential of stem cells, we overexpressed SERPINB2 using a specific retroviral expression vector (Supple. Figure 6). As shown in Fig. 6a, a significant decrease in the number of SERPINB2-overexpressing stem cells was observed compared with the control vector-transfected cells. A flow cytometry assay using Annexin-V was performed to investigate the effect of SERPINB2 overexpression on stem cell apoptosis. The early and late apoptotic rate of MSCs transfected with a SERPINB2-overexpressing vector reached 5.08% and 10.13%, whereas this rate was 0.69% and 3.95% in control vector-transfected MSCs (Fig. 6b), respectively. Consistently, elevated levels of cleaved caspase-3 and PARP (Fig. 6c), and DNA fragmentation (Fig. 6d) were also observed with SERPINB2-overexpressing stem cells. Following SERPINB2 overexpression, the migration potential across the membrane was markedly decreased (Fig. 6e). To further confirm the inhibitory effect of SERPINB2 on the migration of stem cells, western blotting was used to evaluate the levels of MMP-2, which plays a key role in regulating MSC migration. SERPINB2-overexpressing stem cells had significantly decreased MMP-2 expression compared with that of the control vector-transfected cells (Fig. 6f). Moreover, SERPINB2 overexpression markedly decreased multi-lineage differentiation potential toward osteoblasts and adipocytes in vitro (Fig. 6g). Additionally, to further confirm whether SERPINB2 can mediate various stem cell functions, we knocked down SERPINB2 expression using a specific shRNA in stem cells (Supple. Figure 7). Interestingly, SERPINB2 knockdown enhanced growth potential of stem cells (Fig. 7a). Following SERPINB2 knockdown, SERPINB2 knockdown migration potential across the membrane was markedly increased (Fig. 7b). Moreover, SERPINB2 knockdown also enhanced the multi-lineage differentiation potential of stem cells toward osteoblasts in vitro (Fig. 7c). These results suggest that SERPINB2 might play a crucial role in regulating stem cell growth, migration activity, and differentiation potential.

## Discussion

Recently stem cells have received enormous attention as an alternative high-throughput screening platform for safety testing because of their differentiation potential in vitro and to maintain homeostasis^20^. Indeed, embryonic or normal tissue-derived stem cells and the patient’s own adult stem cells can be obtained and differentiated into various target tissues, including cardiomyocytes, hepatocytes, or neurons, for in vitro potential toxicity screening^21^. Especially, MSCs have been used for assessing the toxicity of various chemicals such as anesthetics^22, 23^, chemotherapeutic drugs^24, 25^, endocrine disruptors^26^, nanoparticles^27, 28^, and other industrial chemicals including cosmetic ingredients or detergent^29, 30^. Nevertheless, the comparison among the toxicological studies using MSCs is challenging, since laboratories around the world lack internationally standardized methods for isolation and in vitro expansion of MSCs, resulting in significant functional heterogeneity and inconsistent testing results. To date, only four studies with human MSC have used the neutral red uptake (NRU) assay for the estimation of cell viability or cytotoxicity, as recommended by Interagency Coordinating Committee on the Validation of Alternative Methods (ICCVAM). The cellular heterogeneity of MSCs can be organized and characterized by specific biomarkers. Therefore, the discovery of promising biomarkers for predicting toxicity may overcome the heterogeneity which is one of the key challenges in MSC-based in vitro toxicity screening.

Seeking to define a common marker for predicting stem cell toxicity, we focused on a small group of genes that are functionally related to toxicant-induced hazardous phenotypes in human stem cells. One of these genes, SERPINB2, also known as PAI-2, is significantly enhanced by toxic exposure. SERPINB2 expression was found to be markedly increased in response to multiple “danger” signals, such as bacterial lipopolysaccharide (LPS)^31^, hypoxia^32^, and cytotoxic agents^33, 34^. Moreover, recent studies have illuminated its multiple functions, including roles in cell proliferation, differentiation, and tumorigenesis. Recent studies have shown that enhanced SERPINB2 expression suppresses cell growth and induces the expression of differentiation-specific markers^16^. SERPINB2 expression has also been associated with tumor promotion and poor prognosis in various cancers, such as bladder^35^, colorectal^36^, endometrial^37^, and ovarian^38^ cancers. Additionally, Qin Hu et al. revealed the enhanced expression of SERPINB2 in human keratinocyte caused by exposure to dioxin^39^. These results suggest that SERPINB2 could serve as a sensitive marker for predicting toxic responses to potential hazardous materials. In the current study, we observed increased expression of SERPINB2 after in vitro and in vivo stimulation with toxicant in human UCB-derived stem cells (Fig. 3). Significantly enhanced SERPINB2 expression levels were detected with three of ten tested toxic substances, and moderately increased levels were also were detected with three other substances (Fig. 5). These results warrant further prospective studies to verify the reliability of SERPINB2 as a universal marker for predicting toxicity. The enhanced SERPINB2 expression significantly reduced the self-renewal capacity (Fig. 6a–d), migration ability (Fig. 6e, f), and multi-lineage differentiation potential (Fig. 6g) of human stem cells. These results suggest that SERPINB2 might play a crucial role in regulating stem cell growth, migration activity, and differentiation potential. Interestingly, SERPINB2 overexpression induced significant apoptosis in human stem cells (Fig. 6a–d), but dioxin treatment did not (Supple. Figure 4A-D). This discrepancy could be explained as the result of differences in SERPINB2 expression levels in both conditions. While SERPINB2 overexpression using a specific retroviral expression vector markedly increased mRNA levels of SERPINB2 approximately 45-fold in human stem cells, dioxin exposure stimulated the mRNA levels approximately 6-fold. These differences reflect differences in the activity of SERPINB2 linked to protein production and apoptotic cell death.

Currently, the number of hazardous materials is growing as a result of rapid industrialization, warranting a better screening platform for predicting toxicity. Our findings illuminate the critical requisites for the development of a target gene-based screening platform, namely, the identification of target genes in response to toxic stimuli. We show that the harmful effects of toxic materials on the self-renewal and stem cell-like properties of stem cells can be achieved by maintaining SERPINB2 expression. In conclusion, by combining the toxic-response gene SERPINB2 and human stem cells, we can develop an in vitro screening platform for multiple toxic substances. Furthermore, our stem cell-based screening platforms can provide valuable information on toxic compounds that are not normally detected by other somatic cell-based systems.

## Methods

### Reagents

Aristolochic acid 1 (Cat No: A5512), benzidien (Cat No: B3503), semustine (Cat No: S4026), TPA (Phorbol 12-myristate 13-acetate) (Cat No: P8139), 1,2 dichloropropane (Cat No: 82270), 1,3 butadiene (Cat No: 202533), 4,4 methylene-bis (Cat No: 117323), and carbon tetrachloride (Cat No: 289116) were purchased from Sigma-Aldrich (St. Louis, MO).

### Isolation and culture of human UCB-derived MSCs

Human UCB-derived MSCs were obtained from umbilical vein immediately after delivery with consent from the mother and approval of the Boramae Hospital Institutional Review Board (IRB). Cord blood samples were mixed with HetaSep solution (Stem Cell Technology, Vancouver, BC, Canada) in a ratio of 5:1. MSCs were isolated from other cell types by centrifuging for 20 min at 1200×*g* on a single-density Percoll layer. The cells were washed twice in PBS. Isolated cells were then cultured in growth media consisted of D-media (Formula No. 78-5470EF, Gibco BRL) containing EGM-2 SingleQuot and 10% FBS (Gibco BRL) at 37 °C in humidified atmosphere of 5% CO_2_ in air. After 3 days, non-adherent cells were removed by washing with PBS. All procedures for preparation and utilization of MSCs (IRB no. GAIRB2014-350) for research purposes were approved by the Institutional Review Board of Gachon University.

### Osteogenic differentiation

MSCs were incubated in DMEM high-glucose medium supplemented with 0.1 µM dexamethasone, 10 mM β-glycerophosphate, 50 µM ascorbate, and 10% FBS with or without dioxin; 0.1% (vol/vol medium) DMSO was used as a vehicle control for dioxin. MSCs were grown for 3 weeks, with medium replacement twice a week. Differentiated cells were stained with Alizarin Red S to detect de novo formation of bone matrix. Alizarin Red S in samples was quantified by measuring the optical density (OD) of the solution at 570 nm.

### Adipogenic differentiation

MSCs were incubated in DMEM low-glucose medium supplemented with 500 µM methylxanthine, 5 µg/ml insulin, and 10% FBS with or without dioxin (Sigma-Aldrich Cat No. 48599); 0.1% (vol/vol medium) DMSO was used as a vehicle control for dioxin. MSCs were grown for 3 weeks, with medium replacement twice a week. Lipid droplet formation was confirmed by oil red O staining. Relative quantification of lipid droplet formation was determined by absorbance measurement at 500 nm.

### Cell proliferation assay

The MTT assay was used to determine the cytotoxicity of dioxin, according to the manufacturer’s protocol. MSCs (2 × 10^4^ cells/well) were seeded in 96-well plates. After 24 h of incubation, the cells were treated with an increasing concentration of dioxin for 72 h; 0.1% (vol/vol medium) DMSO was used as a vehicle control for dioxin. The viable cells were measured at a wavelength of 570 nm using a VersaMax microplate reader.

### Flow cytometry

FACS analysis and cell sorting were performed using FACS Calibur and FACS Aria machines (Becton Dickinson, Palo Alto, CA), respectively. FACS data were analyzed using FlowJo software (Tree Star, Ashland, OR). Antibodies to the following proteins were used: APC-conjugated CD44 (BD Bioscience, Cat. 559942, dilution 1/40), PE-conjugated CD133 (MACS; Miltenyi Biotech, Sunnyvale, CA, 130-080-081, dilution 1/40), CD34 (MACS; Miltenyi Biotech, Sunnyvale, CA, 30-081-002,dilution 1/40), CD44 (MACS; Miltenyi Biotech, Sunnyvale, CA, 130-095-180, dilution 1/40), CD45 (MACS; Miltenyi Biotech, Sunnyvale, CA, 130-080-201, dilution 1/40), CD73 (MACS; Miltenyi Biotech, Sunnyvale, CA, 130-095-182, dilution 1/40), CD105 (MACS; Miltenyi Biotech, Sunnyvale, CA, 130-094-941, dilution 1/40), Annexin V (BD Bioscience, Cat. No. 556547, dilution 1/40), and propidium iodide (PI) (Invitrogen, Cat No P3566, dilution 1/40). Add 5 μl of antibody from each dilution into separate sample tubes containing cells (4 × 10^5^). Mix well and incubate cells on ice for 30 min. Wash with 10 ml of medium. The FACS gates were established by staining with an isotype antibody.

### Isolation of mouse adipose tissue-derived MSCs

All of the animal experiments were approved and carried out in accordance with the Institutional Animal Care and Use Committee (IACUC) (LCDI-2013-0031) of the Lee Gil Ya Cancer and Diabetes Institute of Gachon University. Adipose tissue was minced into small pieces, and then digested in DMEM containing 10% FBS and 250 U/ml type I collagenase for 5 h at 37 °C. The digestion mixture was then filtered through a 40-µm cell strainer. Isolated cells were then cultured in growth media consisted of D-media (Formula No. 78-5470EF, Gibco BRL) containing EGM-2 SingleQuot and 10% FBS (Gibco BRL) at 37 °C in humidified atmosphere of 5% CO_2_ in air.

### In vitro cell migration assay

MSCs were plated at 1 × 10^5^ cells/well in 200 μl of culture medium in the upper chamber of Transwell permeable supports (Corning Inc, Corning, NY) with 8.0-μm pore, polycarbonate membrane, 6.5-mm diameter, and 24-well plate format to track migration of MSCs in response to dioxin treatment after 24 h incubation. The cells on the upper surface of the membranes were completely removed by using a cotton swab. Migrated cells on the lower surface of the membranes were fixed with 4% paraformaldehyde for 10 min, stained with hematoxylin (Sigma-Aldrich), and later the number of cells was counted in three randomly selected fields.

### Real-time PCR

Total RNA was extracted using TRIzol reagent (Invitrogen) according to the manufacturer’s protocol. RNA purity was verified by measuring the 260/280 absorbance ratio. The first-strand cDNA was synthesized with 1 μg of total RNA using SuperScript II (Invitrogen), and one-tenth of the cDNA was used for each PCR mixture containing Express SYBR-Green qPCR Supermix (BioPrince, Seoul, South Korea). Real-time PCR was performed using a Rotor-Gene Q (Qiagen). The reaction was subjected to 40-cycle amplification at 95 °C for 20 s, 60 °C for 20 s, and 72 °C for 25 s. The relative mRNA expression of the selected genes was normalized to that of PPIA and quantified using the ΔΔCT method. The sequences of the PCR primers are listed in Supplementary Table 1.

### Protein isolation and western blot analysis

The protein expression levels were determined by western blot analysis as previously described^40^. Cells were lysed in a buffer containing 50 mM Tris, 5 mM EDTA, 150 mM NaCl, 1 mM DTT, 0.01% NP 40, and 0.2 mM PMSF. The concentration of protein was measured with a Protein assay kit (Bio-Rad Laboratories, Hercules, CA, USA) following the manufacturer’s protocol. Samples containing equal amounts of protein (20 μg/25 μl) were separated by sodium dodecyl sulfate-polyacrylamide gel electrophoresis (SDS-PAGE) and then transferred onto nitrocellulose membranes (Bio-Rad Laboratories). The membranes were blocked with 5% skim milk in Tris-buffered saline containing Tween-20 at room temperature (RT). Then, the membranes were incubated with primary anti-β-actin (Abcam, MA, USA, ab189073), SERPINB2 (Abcam, MA, USA, ab47742), caspase-3 (Cell signaling, MA, USA, #9662), PARP (Cell signaling, MA, USA, #9542) antibodies overnight at 4 °C, and then with HRP-conjugated goat anti-rabbit IgG (BD Pharmingen, San Diego, CA, USA, 554021) and HRP goat anti-mouse IgG (BD Pharmingen, 554002) secondary antibodies for 60 min at RT. Antibody-bound proteins were detected using a SuperSignal™ West Pico PLUS chemiluminescent substrate (Thermo Scientific, Cat No. 34080).

### SERPINB2 knockdown and overexpression

Small hairpin RNA (shRNA: accession No. NM_002575) targeting SERPINB2 and scrambled shRNA (shCon) were purchased from Bioneer (Daejeon, South Korea). For efficient SERPINB2 transfection, reverse transfection was performed using Lipofectamine 2000 (Invitrogen) according to the manufacturer’s protocol. Briefly, shRNA targeting SERPINB2 (3 μg/ml) was mixed with 3 μl transfection reagent lipofectamine 2000 in Gibco opti-MEM media without FBS and antibiotics; 5 h before transfection, opti-MEM was replaced with fresh EGM-2 medium with 10% FBS. We chose the SERPINB2 shRNA that is most effective in mRNA levels from three shRNA designed from the target sequence and determined by qRT-PCR analysis. MSCs were also transfected by SERPINB2 expression vector (Applied biological materials, Richmond, Canada, LV301150) for SERPINB2 overexpression. Transfection was performed by Lipofectamine 2000 (Invitrogen) according to the manufacturer’s protocol. Briefly, SERPINB2 expression vector (3 μg/ml) was mixed with 3 μl transfection reagent lipofectamine 2000 in Gibco opti-MEM media without FBS and antibiotics; 5 h before transfection, opti-MEM was replaced with fresh EGM-2 medium with 10% FBS.

### DNA Microarrays and RNA sequencing analysis of dioxin-treated stem cells

Total RNA from dioxin-treated MSCs and non-treated MSCs (0.1%, vol/vol medium, DMSO) was isolated using Trizol reagent (Invitrogen) and purified with RNeasy columns (Qiagen, Valencia, CA, USA) according to the manufacturer’s instructions. DNA microarrays were performed on the human probe Illumina BeadArray HT-12 (Illumina Inc., San Diego, CA, USA), which covers 34,598 probes. Arrays were scanned using an Illumina BeadStation 500 (Illumina Inc., San Diego, CA, USA). Illumina BeadStudio version 2 software (Illumina Inc., San Diego, CA, USA) was used to generate signal intensity values from the scans. RNA integrity for RNA sequencing was confirmed by a bioanalyzer using an Agilent RNA 6000 Pico Kit (Agilent, Santa Clara, CA). The isolated total RNA was processed for preparing mRNA sequencing library using TruSeq stranded mRNA sample preparation kit (illumina, San Diego, CA) according to manufacturer’s instruction. Three mRNA samples from each group were pooled together in equal concentrations in one pool prior to sequencing. Simply, mRNAs were isolated from 400 ng total RNA by RNA purification bead using polyA capture, and followed by enzyme shearing. After first and second strand cDNA synthesis, A-tailing and end repair were performed for ligation of proprietary primers that incorporate unique sequencing adaptors with index for tracking illumina reads from multiplexed samples run on a single sequencing lane. For each library, an insert size of approximately 270 bp was confirmed by a bioanalyzer using an Agilent RNA 6000 Pico Kit (Agilent, Santa Clara, CA) and quantification of library was measured by real-time PCR using CFX96 real time system (BioRad, Hercules, CA). Sequencing of each library was performed on an illumina NextSeq500 and Clusters of the cDNA libraries were generated on a high-output flow cell and sequenced for 75-bp paired end reads (2 × 75) with a NextSeq 500 High Output 150 cycle kit (illumina, San Diego, CA). The raw image data were transformed by base-calling into sequence data and stored in FASTQ format.

### Statistical analysis

The results are expressed as the mean ± the standard deviation (SD) of at least three independent experiments. The comparisons between the experimental groups and the corresponding controls were performed with GraphPad Prism 5.0 (GraphPad Software, San Diego, CA) using one-way ANOVA. *P* < 0.05 was considered to indicate statistical significance.

## Electronic supplementary material

Supplementary figure 1

Supplementary figure 2

Supplementary figure 3

Supplementary figure 4

Supplementary Figure 5

Supplementary Figure 6

Supplementary Figure 7

supplementary figure legends
