# Supplementary figures and images for: SERPINB2 is a novel indicator of stem cell toxicity
Source: Cell Death Dis. 2018 Jun 20;9(7):724. doi: 10.1038/s41419-018-0748-x (PMC6010432; doi:10.1038/s41419-018-0748-x)

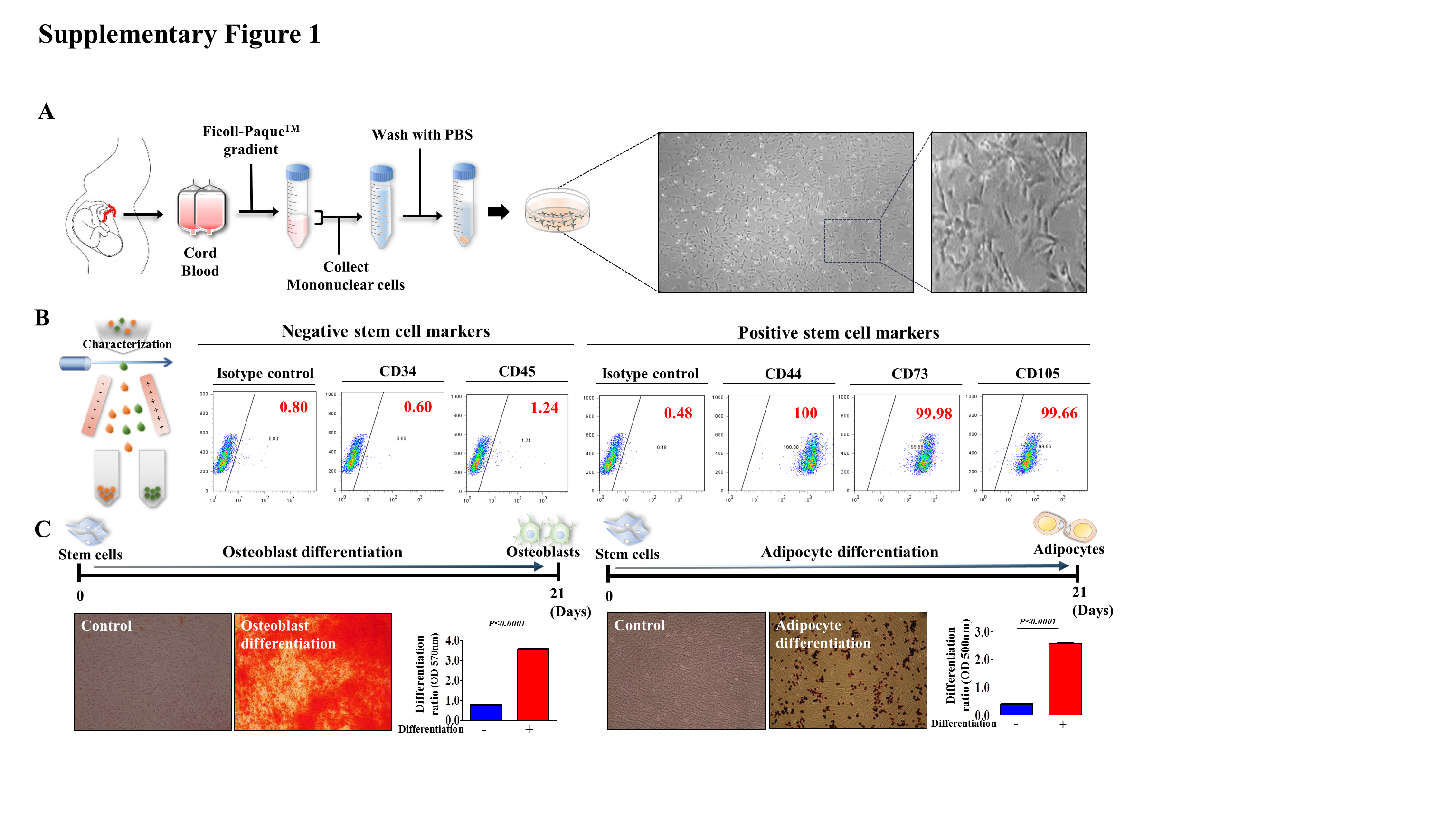

Supplement: Supplementary file 1 — Supplementary figure 1 [file 41419_2018_748_MOESM1_ESM.tif]

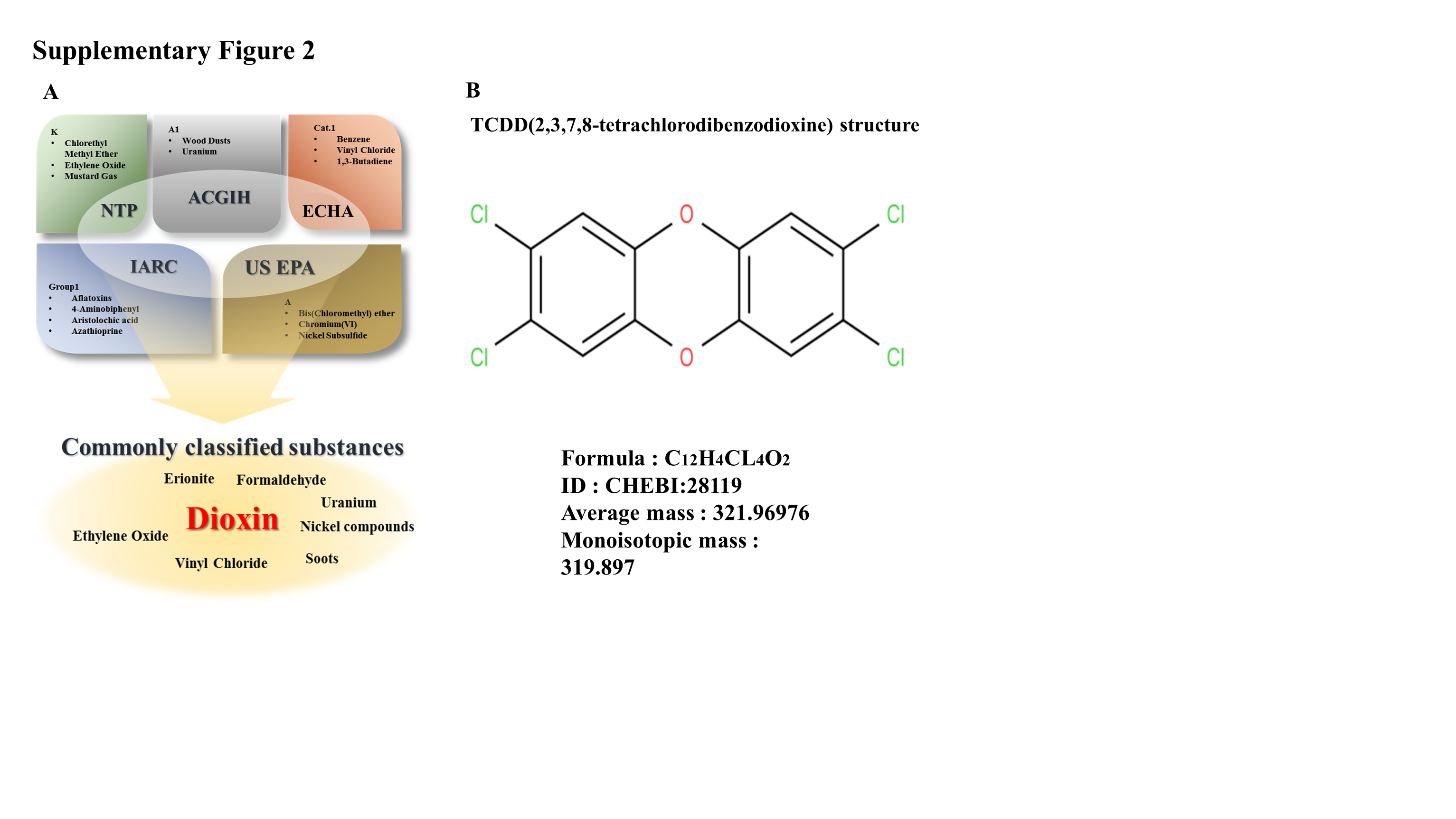

Supplement: Supplementary file 2 — Supplementary figure 2 [file 41419_2018_748_MOESM2_ESM.tif]

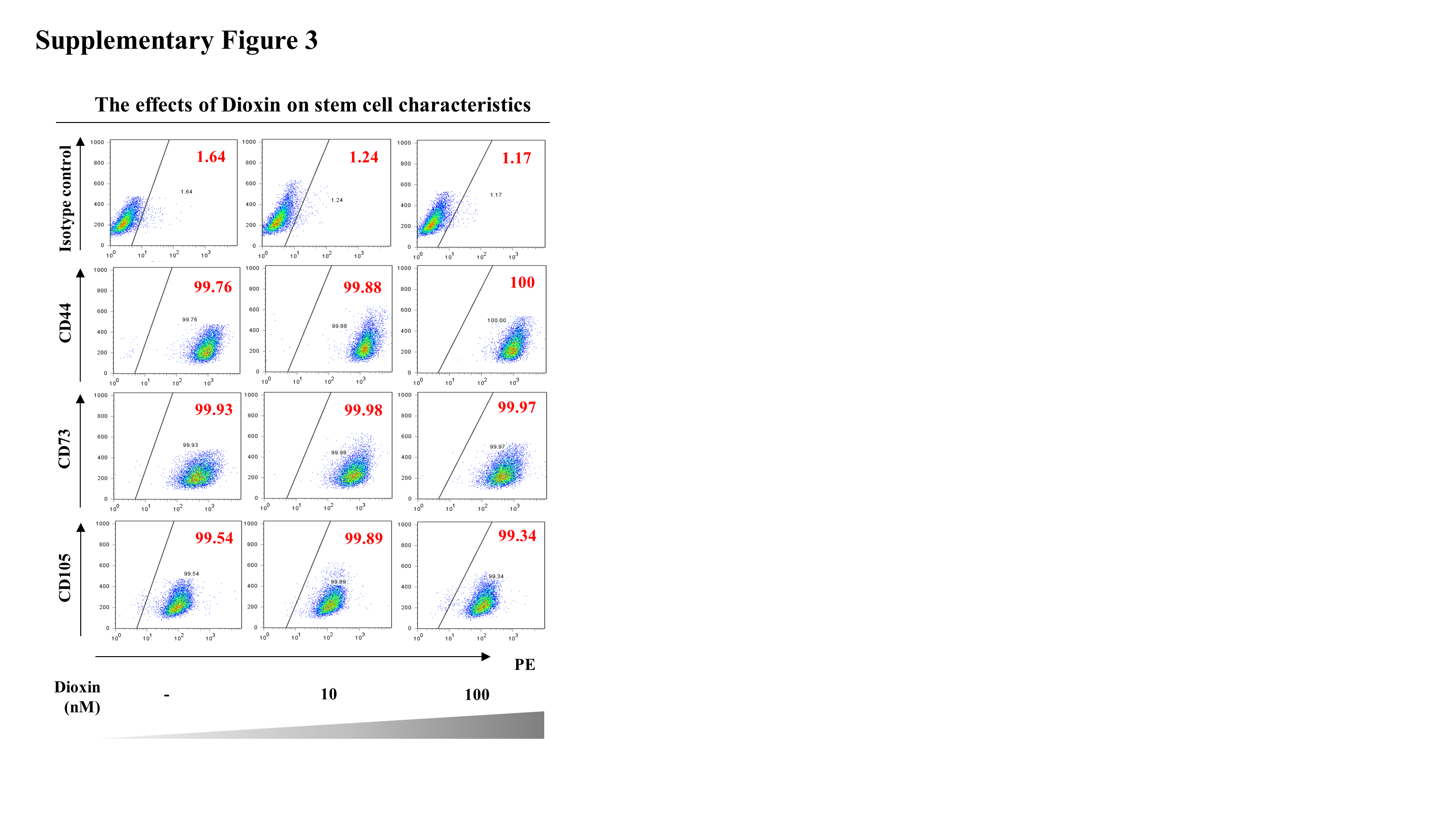

Supplement: Supplementary file 3 — Supplementary figure 3 [file 41419_2018_748_MOESM3_ESM.tif]

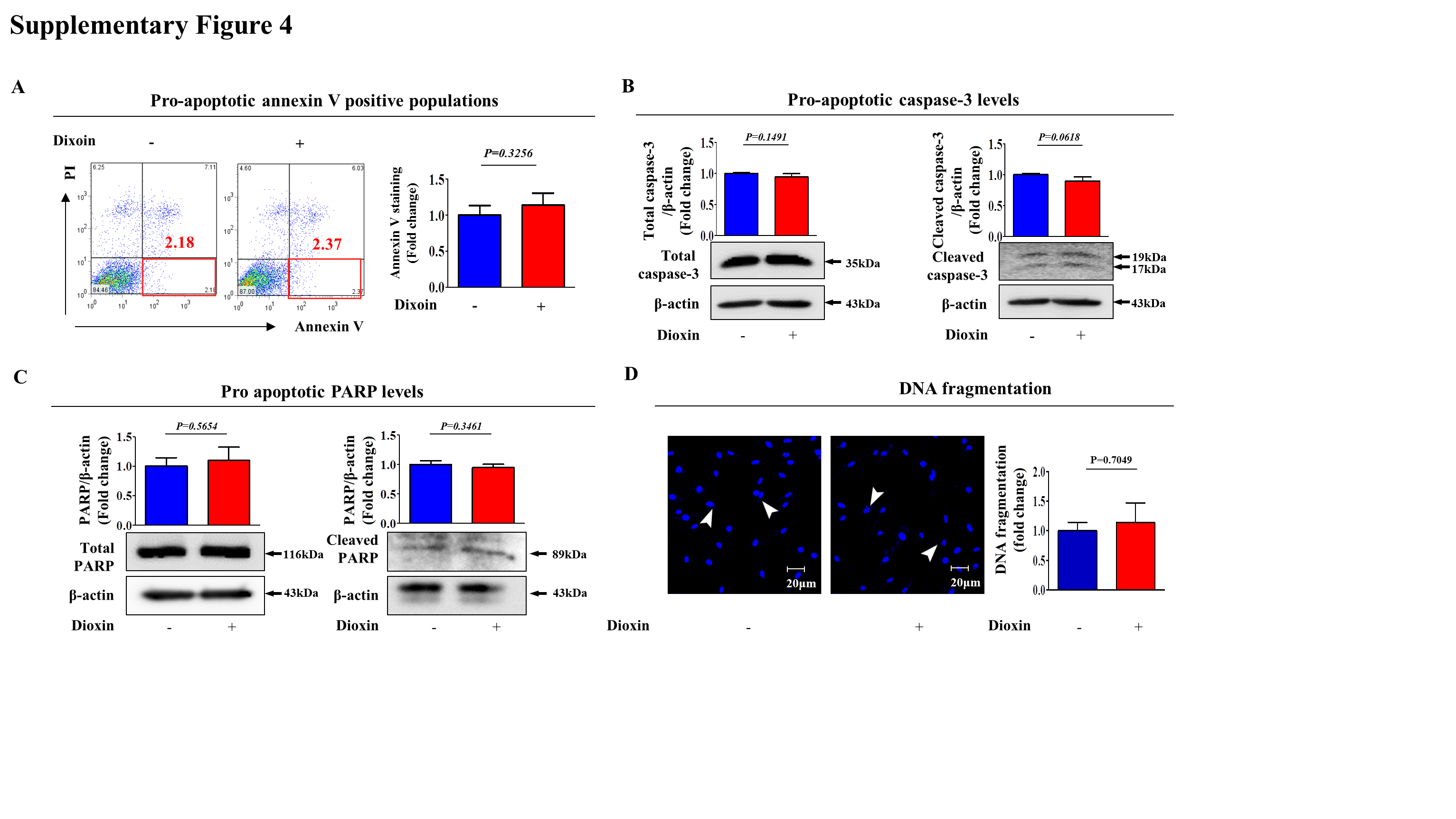

Supplement: Supplementary file 4 — Supplementary figure 4 [file 41419_2018_748_MOESM4_ESM.tif]

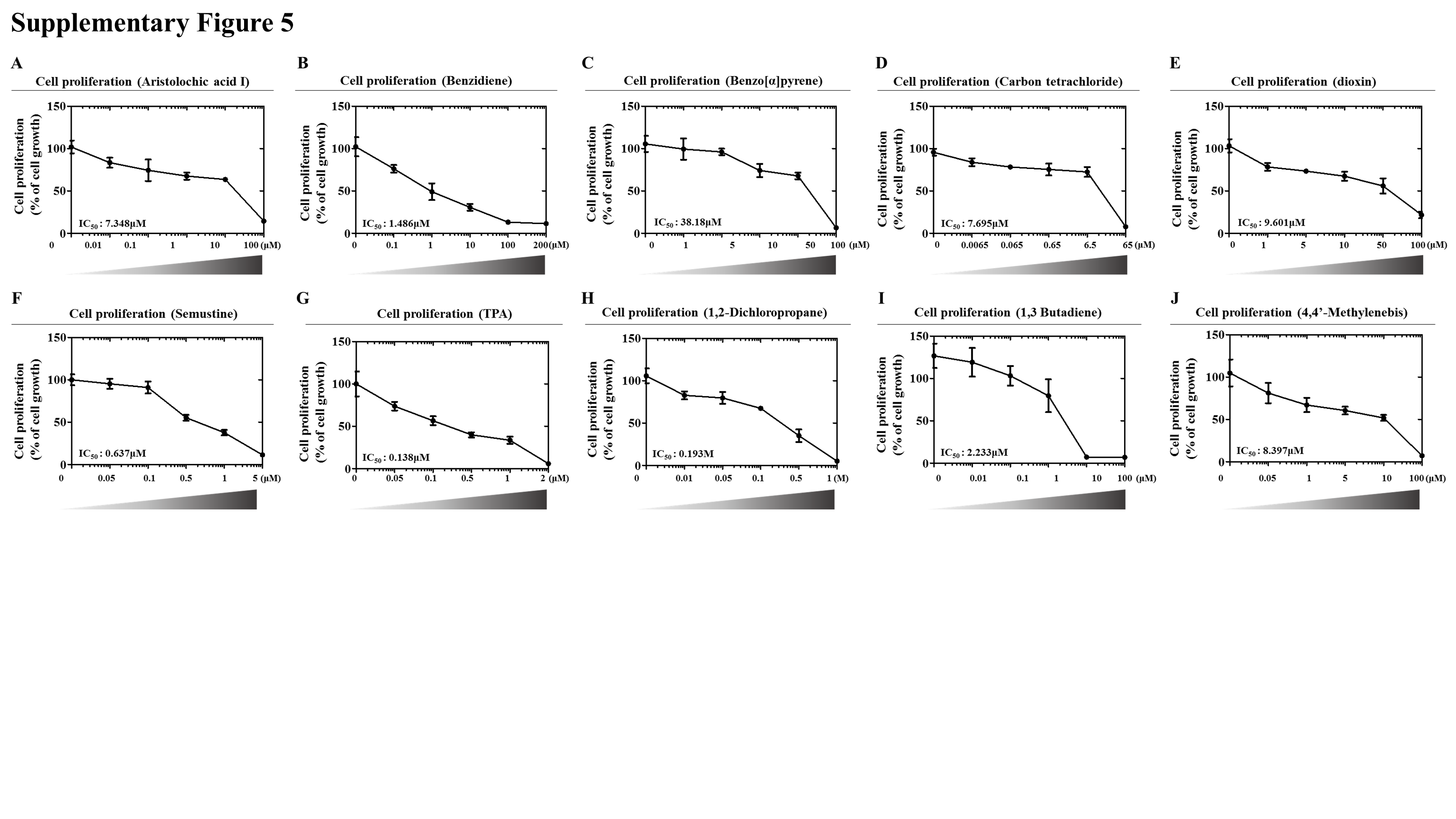

Supplement: Supplementary file 5 — Supplementary Figure 5 [file 41419_2018_748_MOESM5_ESM.tif]

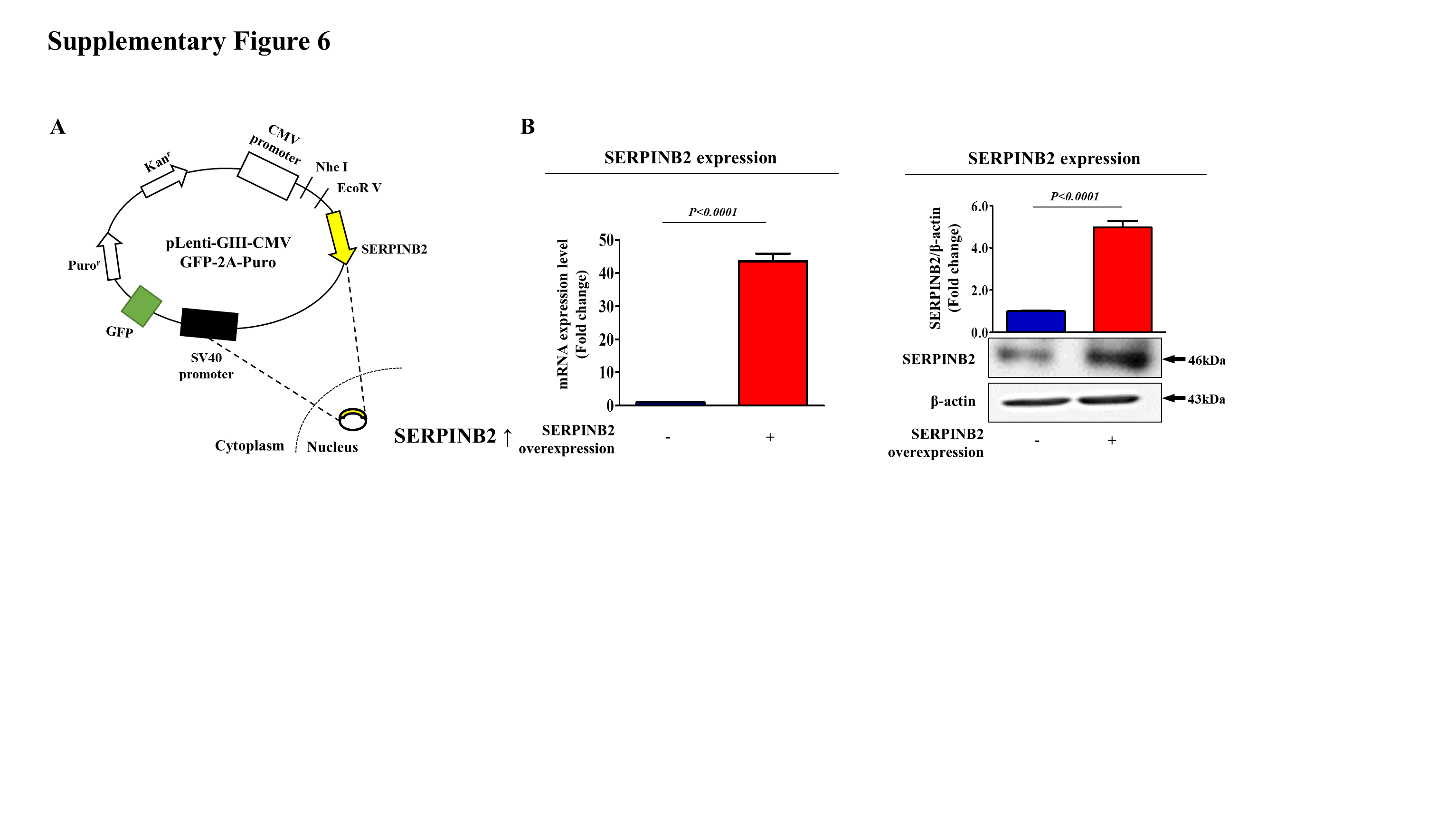

Supplement: Supplementary file 6 — Supplementary Figure 6 [file 41419_2018_748_MOESM6_ESM.tif]

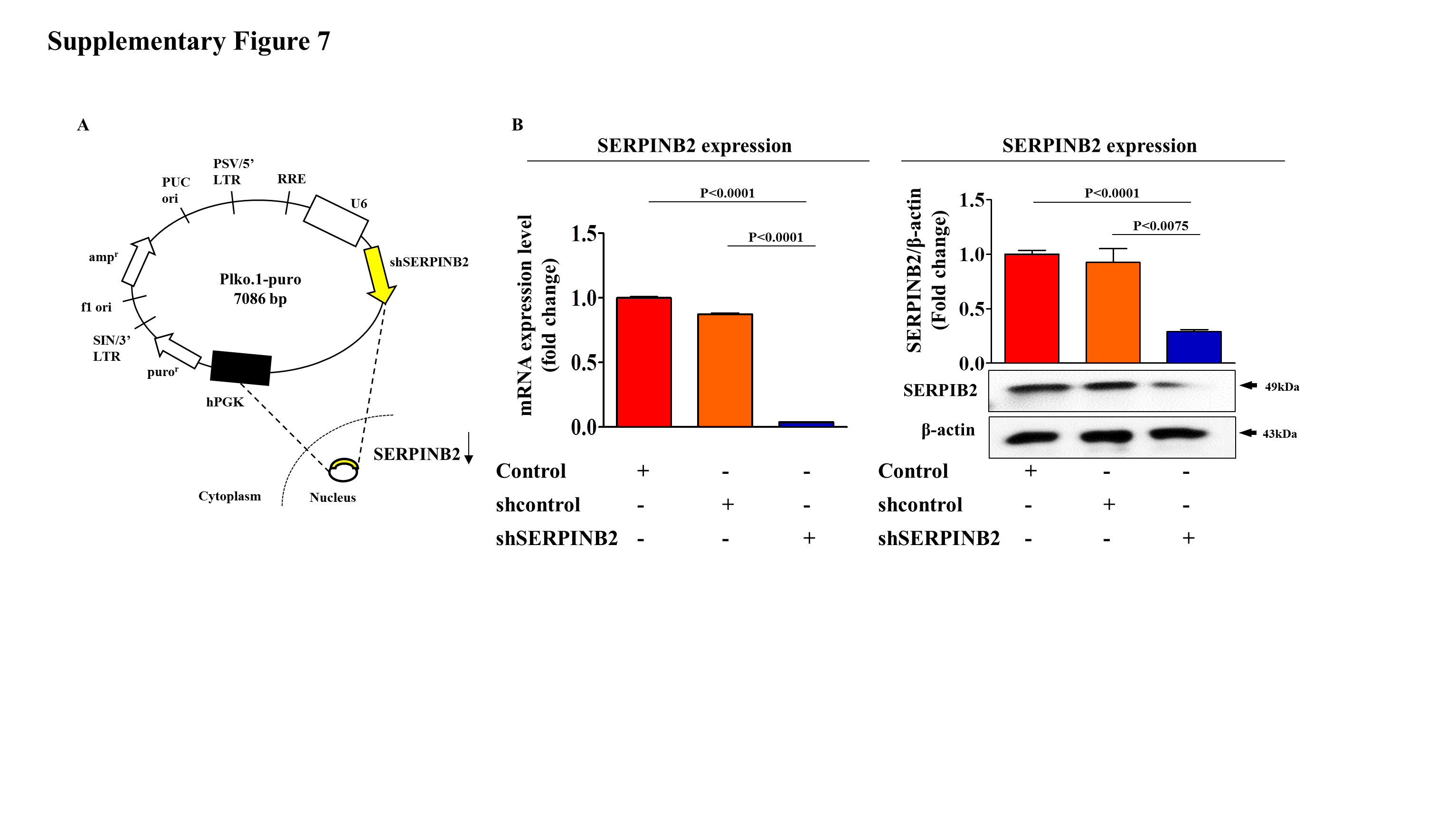

Supplement: Supplementary file 7 — Supplementary Figure 7 [file 41419_2018_748_MOESM7_ESM.tif]
