# Supplementary material for: SERPINB2 is a novel indicator of stem cell toxicity
Source: Cell Death Dis. 2018 Jun 20;9(7):724. doi: 10.1038/s41419-018-0748-x (PMC6010432; doi:10.1038/s41419-018-0748-x)
Supplement: Supplementary file 8 — supplementary figure legends [file 41419_2018_748_MOESM8_ESM.docx]

**Supplementary figure legends**

**Supplementary Figure 1. Isolation and characterization of umbilical cord blood-derived mesenchymal stem cells.** Schematic representation of the experimental protocol as described in the materials and methods section. Spindle-shaped umbilical cord blood-derived MSCs can be observed under phase contrast microscopy **(A)**. The immunophenotypic differentiation patterns of MSCs were analyzed by flow cytometry. The cells expressed putative MSC markers CD44, CD73, CD105 and were negative for the hematopoietic markers CD34 and CD45 **(B)**. The differentiation potential of MSCs into osteoblasts and adipocytes were determined by alizarin red or oil red O staining. Relative quantification of the calcium mineral content or lipid droplet formation was determined by absorbance measurements at 570 nm or 500 nm, respectively **(C)**. The results represent the means ± SD from three independent experiments.

**Supplementary Figure 2. Selection of standard toxic compound (dioxin) for stem cell toxicity.** A standard test compound was chosen from the list of top-ranked compounds according to the common hazardous material classification of five authorized international organizations, including the IARC (International Agency for Research on Cancer), ACGIH (Association Advancing Occupational and Environmental Health), NTP (National Toxicology Program), US EPA (Environmental Protection Agency), and ECHA (European Chemicals Agency). Of the top-ranked hazardous materials, we selected dioxin as a standard test compound due to its severe toxicity and tumorigenicity **(A)**. Chemical information of 2,3,7,8-tetrachlorodibenzodioxin generated from PubChem website. PubChem (https://pubchem.ncbi.nlm.nih.gov/) is open-source chemical database providing chemical structure, chemical formula, and molecular weight. IUPAC Name: 2,3,7,8-tetrachlorodibenzo-p-dioxin; InChI=1S/C12H4Cl4O2/c13-5-1-9-10(2-6(5)14)18-12-4-8(16)7(15)3-11(12)17-9/h1-4H; InChI Key: HGUFODBRKLSHSI-UHFFFAOY SA-N; Molecular formula: [C_12_H_4_Cl_4_O_2_](https://pubchem.ncbi.nlm.nih.gov/search/#collection=compounds&query_type=mf&query=C12H4Cl4O2&sort=mw&sort_dir=asc). Molecular weight: 321.962 g/mol **(B)**.

**Supplementary Figure 3. The effects of dioxin on the various markers of stem cells *in vitro*.** The effects of dioxin treatment (10 nM) on the expression pattern of the putative stem cell markers (CD44, CD73, and CD105) were analyzed by flow cytometry.

**Supplementary Figure 4. Effects of dioxin treatment on the apoptosis of stem cells.** Dioxin-induced cytotoxicity was evaluated by flow cytometry using PE-labeled Annexin-V **(A)**. The fragment levels of caspase-3 and PARP following dioxin treatment were assessed by western blotting **(B-C)**. Dioxin-mediated apoptotic DNA fragmentation and condensation were visualized using DAPI staining **(D)**. β-actin was used as an internal control. DAPI staining was used to label the nuclei within each field. The results represent the means ± SD from three independent experiments.

**Supplementary Figure 5. The treatment concentration that inhibits 50% of the cell proliferation (IC_50_).** The inhibition of cell viability using multiple test substances, such as aristolochic acid I (7.348 µM), benzidine (1.486 µM), benzo[a]pyrene (38.18 µM), Carbon tetrachloride (7.695 µM), dioxin (9.601 µM), semustine (0.637 µM), TPA (0.138 µM), 1,3-butadiene (2.233 µM), 1,2-dichloropropane (193 mM), and 4,4-methylenebis (8.397 µM), for 48 hours was determined using an MTT assay in stem cells from three independent experiments **(A-J)**. The cell viability (%) was calculated as the percent of the vehicle control.

**Supplementary Figure 6. Overexpression efficiency of a SERPINB2-expressing vector.** MSCs were stably transduced with a retroviral vector overexpressing SERPINB2. The successful overexpression of SERPINB2 was verified based on the RNA and protein levels in stem cells **(A-B)**. β-actin was used as the internal control. The results represent the mean ± SD from three independent experiments.

**Supplementary Figure 7. Knockdown efficiency of SERPINB2 in stem cells.** MSCs were stably transduced with shRNA which target SERPINB2, or with a non-targeting control shRNA **(A)**. The successful knockdown of SERPINB2 was verified based on the RNA and protein levels in stem cells **(B)**. β-actin was used as the internal control. The results represent the mean ± SD from three independent experiments.
